# Cancer of the Skin

**DOI:** 10.1038/sj.bjc.6602578

**Published:** 2005-05-17

**Authors:** I M Leigh

**Affiliations:** 1 Barts & the London, Queen Mary's School of Medicine & Dentistry, University of London, London, UK

This substantial tome is a comprehensive update of a text published 10 years ago and covers a wide range of issues in skin cancer from clinical and epidemiological studies through to detailed cellular biology. The six eminent editors are joined by a cast of over a hundred co-authors, which include many very senior established international figures in the fields of both melanoma and non-melanoma skin cancer. This group leans very heavily on American contributions and there are few European contributors. This diversity is both a strength and weakness. The text is certainly comprehensive, but as each chapter is self-contained there is considerable overlap and repetition. There are also some idiosyncrasies: there is a chapter on Bowenoid papulosis for example, which is accepted by those working in vulval dermatology to be vulval intraepithelial neoplasia. The photographic illustrations are of very high quality and add significantly to the value of the book and there are many constructive diagrams, especially in the excellent chapter on the biology of the melanocyte by Schaffer and Bolognia.

The book has seven parts, which do not always appear systematic or logical in their content nor balanced in their size and structure. The first section entitled Basic Cancer Biology and Epidemiology, for example, covers the biology of invasion and metastasis before essential cancer biology in chapters on the genetic and cellular basis of skin carcinogenesis. This is followed by the basic cell biology of the melanocyte, but there is no chapter on keratinocyte biology (keratinocyte differentiation, etc.). Given the involvement by Robin Marks, who has been instrumental in cancer prevention in Australia, there is an understandable emphasis on prevention and use of sunscreens.

Part two focuses on non-melanoma skin cancer and it is suggested that these should be called keratinocyte malignancies (rather than something they are not!). This is entirely sensible, but given the acceptance of the terminology as basal cell and squamous cell carcinomas in clinical practice, unlikely to catch on. Precursors to these lesions are largely squamous in nature; so a dedicated chapter could usefully have been integrated with the chapter on SCCs, particularly where therapy is considered. Vulval intraepithelial neoplasia merits a more comprehensive approach and perhaps epidermodysplasia verruciformis could have been considered with other genetic diseases, as although this contribution is clinically comprehensive for this extremely rare genodermatosis, there is little virology included and genetic syndromes are covered in part four. The third part dedicated to melanoma is very comprehensive but has a superfluity of clinical photographs, particularly of naevi. The diagnosis and role of dysplastic naevi and giant congenital naevi are very thoroughly described in dedicated chapters. Genetic testing for p16 is rightly recommended to be undertaken for research purposes, but still covered with consent forms in a format acceptable to the American Society for Clinical Oncology, but not necessarily to other groups working in the field. Management of surgical options and adjuvant therapy are described, but there is also a short discussion of the management of metastatic disease here.

There are a large number of unusual and rare cancers of the skin, such as sarcomas of the skin, adnexal tumours and Merkel cell carcinoma, which are very fascinating to dermatologists and dermato-pathologists, but perhaps not often diagnosed at clinical presentation. Part four contains chapters, not only on these tumours, but also the clinically much more important T-cell lymphomas and pseudo-lymphomas, which merit much greater attention than the short exposition included. A concise discussion of genetic syndromes predisposing to malignancy includes basal call naevus syndrome, xeroderma pigmentosum and epidermolysis bullosa. However, the opportunity to discuss in detail the molecular mechanisms which are so important: DNA repair in XP, for example, where there has been a solid contribution to understanding DNA repair mechanisms is not included, which seems an omission in a book of this size and complexity. There is also repetition and overlap with the previous chapter on the genetic basis of skin cancer. A chapter entitled ‘Skin markers of internal malignancy’ confirms the suspicion that this book is highly oriented towards dermatologists rather than oncologists. A short fifth section on diagnosis includes dermoscopy, again a superfluity of photographs repeating previous chapters; computer analysis as an aid to diagnosis of melanoma and reflectance confocal microscopy, a technique not yet enjoying widespread acceptance. A substantial sixth section covers the many established modes of treatment – curettage and electrodessication, cryotherapy and surgical excision, as well as the newer techniques using photodynamic therapy and immune response modifiers. Melanoma treatment is here thoroughly explored with sentinel node biopsy being prominent, but also reconstructive surgery. A chapter on adjuvant therapy is particularly thorough and treatment of metastatic disease overlaps a little with the section on melanoma. Reconstructive surgery describing particularly flap grafts might sit better in a more comprehensive surgical text for plastic and reconstructive surgery. The final small section includes tanning, photography and medico-legal issues.

This book will be of potential interest to dermatologists, the relevance to pathologists or oncologists being restricted to certain subjects and chapters scattered through the book. It is particularly interesting for those dermatological practitioners in this field to read the forceful views of eminent dermatologists, but repetition makes it irritating to synthesise a consensus. The growing importance of skin cancer requires widespread dissemination of the information about the importance of skin cancers, their rapidly rising incidence and diagnostic and therapeutic aspects of clinical practice, and this book certainly covers the field. However, it is likely to be more of a reference book for educational purposes rather than for clinical practice, and would have benefited from stricter editing with a reduction of overlap and a smaller number of contributors so that there is a more coherent style. It will no doubt be found on the shelf of those with a clinical interest in skin cancer.

